# Raman Spectroscopic Study of Amyloid Deposits in Gelatinous Drop-like Corneal Dystrophy

**DOI:** 10.3390/jcm11051403

**Published:** 2022-03-04

**Authors:** Giuseppe Acri, Antonio Micali, Rosalia D’Angelo, Domenico Puzzolo, Pasquale Aragona, Barbara Testagrossa, Emanuela Aragona, Edward Wylegala, Anna Nowinska

**Affiliations:** 1 Department of Biomedical Sciences, Section of Physics, University of Messina, 98125 Messina, Italy; giuseppe.acri@unime.it (G.A.); barbara.testagrossa@unime.it (B.T.); 2 Department of Adult and Pediatric Pathology, University of Messina, 98125 Messina, Italy; 3 Department of Biomedical Sciences, Section of Biology and Genetics, University of Messina, 98125 Messina, Italy; rosalia.dangelo@unime.it; 4 Department of Biomedical Sciences, Section of Histology and Embryology, University of Messina, 98125 Messina, Italy; puzzolo@unime.it; 5 Department of Biomedical Sciences, Eye Clinic, Regional Referral Center for the Ocular Surface Diseases, University of Messina, 98125 Messina, Italy; paragona@unime.it; 6 Department of Ophthalmology, Scientific Institute San Raffaele, Vita-Salute University, 20132 Milan, Italy; emanuela.aragona25@gmail.com; 7 Chair and Clinical Department of Ophthalmology, Faculty of Medical Sciences in Zabrze, Medical University of Silesia, 40-555 Katowice, Poland; ewylegala@sum.edu.pl (E.W.); atrum2@gmail.com (A.N.); 8 Ophthalmology Department, Railway Hospital, 40-760 Katowice, Poland

**Keywords:** cornea, gelatinous drop-like corneal dystrophy, *TACSTD2* gene mutation, amyloid deposits, Raman spectroscopy, light microscopy

## Abstract

The genetic and histopathological features of the cornea of a Polish patient with Gelatinous Drop-like Corneal Dystrophy (GDCD) and the molecular composition with Raman spectroscopy of corneal deposits were examined. A 62 year-old Polish woman was diagnosed with GDCD and underwent penetrating corneal transplant. A blood sample was collected, and genetic analysis was performed. The cornea was processed for light microscopy and Raman analysis. The genetic exam revealed a previously undescribed homozygous 1-base pair deletion in exon 1 of *TACSTD2* gene (c.185delT), resulting in a frame shift causing a premature stop codon. When compared with a control cornea, in GDCD cornea stained with PAS evident deposits were present over the anterior stroma, with apple green birefringence under polarized light. Raman spectroscopy showed peculiar differences between normal and GDCD cornea, consisting in peaks either of different height or undetectable in the normal cornea and related to amyloid. The possible causative role of the novel mutation was discussed and Raman spectroscopy as a further morphological tool in the evaluation of corneal dystrophies, characterized by the deposition of abnormal materials, was suggested.

## 1. Introduction

The cornea, a transparent barrier between the external environment and the inner structures of the eye, provides a smooth surface for light refraction and immunologic protection [1]. It consists of several layers: from the surface, the epithelium, the Bowman’s layer, the stroma, the Descemet’s membrane, and the endothelium [2].

Corneal transparency can be altered by the deposition of abnormal material, as in corneal dystrophies (CD), characterized by a genetic etiology, either inherited or caused by a de novo mutation [3].

Gelatinous Drop-like Corneal Dystrophy (GDCD; Online Mendelian Inheritance in Man #204870) is a rare CD [3,4], whose clinical symptoms are photophobia, tearing, corneal foreign body sensation, and severe progressive loss of vision [5,6], owing to the accumulation of milky-white gelatinous mulberry-shaped excrescences under the corneal epithelium. It is an autosomal recessive inherited disorder, included in category 1 according to the International Committee for Classification of Corneal Dystrophies (IC3D), as the involved gene has been identified and mapped, and specific mutations are known [7]. In fact, GDCD locus was mapped to chromosome 1p32 [8]. GDCD was linked to mutations in the tumor-associated calcium signal transducer 2 (*TACSTD2*) gene, which encodes for TACSTD2, a human transmembrane glycoprotein, essential for the integrity of the corneal epithelial barrier [9]. In GDCD, the dysfunction of TACSTD2 interferes with tight junction proteins claudin-1 and -7, causing the penetration of lactoferrin, lysozyme, and other molecules from the tears and their deposition as amyloid deposits in the stroma [10,11]. Histologically, acellular deposits able to raise or interrupt the Bowman’s layer were observed in subepithelial and anterior stromal location. Amyloid deposits showed a brick red stain with Congo red stain and displayed an apple green birefringence under polarized light [6,12].

Raman spectroscopy is an inelastic light-scattering phenomenon providing vibrational spectra containing information on the chemical bonds and the symmetry of a specific molecule [13,14,15,16]. Raman spectroscopy is considered as the “fingerprint” of material identification [17], as it can reveal molecular structures and discriminate between different substances. For these characteristics, it appears particularly useful in the comparison between normal and pathological tissues or cells, representing a valid tool in clinical diagnosis [18,19,20,21,22].

In ophthalmology, Raman spectroscopy was used to evaluate the composition of tears [23,24], vitreous [25], and aqueous humor [26], and to monitor the evolution of experimental retinal inflammation [27]. Furthermore, the biochemical characteristics of cultured mouse keratocytes in experimental keratomycosis [28], of the cornea in nephropathic cystinosis [29], and of the normal rat cornea [30] have been examined.

While clinical, genetic, and morphological data of GDCD cornea are present in the literature [4,6,11,12], no biophysical studies, as to our knowledge, are currently available on GDCD cornea ex vivo with Raman spectroscopy.

Aim of the present paper was to consider the role of our GDCD patient’s genetic profile in the behavior of corneal biomolecules and to provide clinicians with valuable information to be translated in an in vivo assessment of corneal composition in ophthalmic diseases.

## 2. Materials and Methods

### 2.1. Ethical Approval

The Ethics Committee of the Medical University of Silesia in Katowice, Poland approved the use of tissue evaluation (KNE/0022/KB1/43/I/14; 1 July 2014). All procedures were conducted according to the tenets of the Declaration of Helsinki. Informed consent was obtained from the subject, after explanation of the nature of the study.

### 2.2. Clinical Data

A 62 year-old female patient was affected with GDCD from early childhood. In 2015, a penetrating corneal transplant was performed in the left eye; after the transplant, significant problems related to reduced epithelialization, ocular surface severe inflammation, glaucoma, and recurrence of CD were demonstrated, which led in two years to corneal leucoma and edema with hand motion visual acuity.

**Figure 1:**
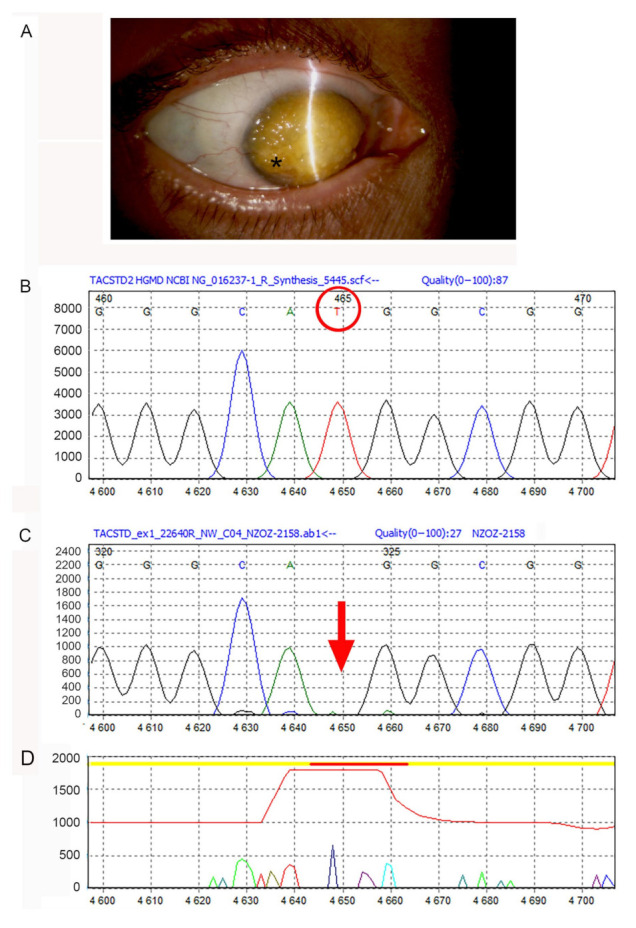
Raman spectroscopy in a corneal dystrophy. (**A**) Slit lamp photograph of the right eye, showing corneal deposits with the typical mulberry-like appearance and peripheral neo-vascularization (*). (**B**–**D**): Electropherogram of the c.185delT mutation of the TACSTD2 gene (Sanger sequencing and analysis by Mutation Surveyor 4.0 software) detected in our patient. (**B**) Control DNA. The red circle indicates the thymine (T) base, which lacks in the patient’s DNA. (**C**) Patient’s DNA. Arrow = c.185 mutation. (**D**) Difference of the sequencing raw data between control and patient.

In 2019, she was referred to the Chair and Clinical Department of Ophthalmology of the Medical University of Silesia, Katowice, Poland, where she was diagnosed with a mulberry-like GDCD cornea with peripheral neo-vascularization (Figure 1A). Therefore, she underwent a penetrating corneal transplant in the right eye. The last recorded visual acuity was 0.01, using the decimal unit system. On slit lamp, multiple corneal small erosions and corneal edema were present. The corneal button was processed for light microscopy and Raman spectroscopy.

### 2.3. Genetic Analysis

Blood sample was collected from the patient after obtaining written informed consent for genetic testing. Genomic DNA was extracted from blood sample using MagCore Genomic DNA Whole Blood Kit (RBC Bioscience Corp., New Taipei City, Taiwan). A targeted exome sequencing was performed on the following genes linked with corneal dystrophies: *CHST6, TGFBI, KRT3, KRT12, COL8A2, SLC4A11, TACSTD2, UBIAD1, VSX1,* and *ZEB1*. Targeted Exome panel sequencing was performed with the SeqCap EZ Hyper Cap protocol and a NimbleGen SeqCap EZ probe kit (Roche Sequencing Solutions, Inc; CA; USA) using a NextSeq 550Dx sequencer by Illumina (Illumina, San Diego, CA, USA) in 2 × 150 paired-end sequencing mode. The targeted region presented a mean coverage of 418× with 100% of targeted bases covered at least 40x. Validation of the called variants was performed by Sanger sequencing and specific flanking intronic primer pairs were designed using Primer3web (https://primer3.ut.ee/, accessed on 4 January 2022) and NCBI Primer-BLAST (https://www.ncbi.nlm.nih.gov/tools/primer-blast/, accessed on 4 January 2022), spanning a fragment of the 5′UTR region and a fragment of exon 1 (primers are available upon request). For the analysis of the *TACSTD2* gene, the reference sequence with accession number NM_002353.2 (HGMD Professional 2019.4) was used. All variants were described in concordance to the Human Genome Variation Society nomenclature (HGVSv19.01).

### 2.4. Light Microscopy

The uninvolved part of the cornea of a subject who underwent penetrating keratoplasty for keratoconus was used as control. As the specimen was acquired form a surgical treatment, only informed consent was obtained, after explanation of the nature of the study. Both GDCD and control cornea were fixed in 4% paraformaldehyde in phosphate buffer pH 7.4 for 24 h, dehydrated with ethanol, cleared with xylene, and embedded in paraffin (Paraplast; Sigma-Aldrich, Milan, Italy). Five μm sections were cut using a Leica RM2125 microtome (Leica Instruments, Nussloch, Germany), placed on poly L-lysine coated slides, dewaxed with xylene, rehydrated in ethanol, and stained with PAS and Congo Red stains. In order to avoid differences related to the technical procedures between the control and the pathological cornea, we processed them at the same time, using the same reagents at the same concentrations, when necessary, and for the same times. Three samples for each cornea were photographed with a Nikon Ci-L (Nikon Instruments, Tokyo, Japan) light microscope using a digital camera Nikon Ds-Ri2 and saved as Tagged Image Format Files (TIFF) with the Adobe Photoshop CS software. For the polarized light microscopy, a simple orthoscopic polarized light microscope Nikon NiU with 20 X Plan Apochromat Lambda (Nikon Instruments, Tokyo, Japan) objective was used.

### 2.5. Raman Spectroscopy Measurements

All the Raman measurements were performed with a DXR-SmartRaman Spectrometer (Thermo Fisher Scientific, Waltham, MA, USA) using a diode laser with the excitation wavelength of 785 nm. All Raman spectra were acquired over the wavenumber range of 3300–400 cm^−1^, with a resolution of 1.9285 cm^−1^ and irradiated with a laser power of 24 mW, coming out from a 50 µm spot. Before conducting measurements, a calibration with standard samples of known wavenumber was performed. Normal and GDCD corneal sections of 40 μm were cut as above indicated, dewaxed in xylene, and air-dried; then they were placed into the sample holder and the 180 Degree Sampling Accessory was used for measurements. Raman spectrum of the cornea was obtained as 32 replicates of each sample, and every replicate spectrum was obtained with an acquisition time of 60 s. The final spectrum is the average of the 32 exposures performed and it is automatically showed by the built-in software of the spectrometer. The spectrum was obtained from the anterior part of the cornea stroma, where the deposits (as demonstrated by the histological micrographs) are more numerous. All the Raman spectra were stored in .SPA format and the post processing analysis was performed using the Omnic for dispersive Raman 9.0 software. Four different acquisitions were performed on each cornea sample (three samples), the obtained spectra showed no statistical differences. To obtain adequate information from acquired spectra, we performed a baseline correction of each of them, in order to compensate eventual technical and/or sample variations, and we normalized them to the phenylalanine band, located near 1003 cm^−1^, as it has been demonstrated to be insensitive to the micro-environment [31].

## 3. Results

### 3.1. Genetic Analysis Data

A homozygous deletion in exon 1 of the *TACSTD2* gene, c.185delT (p.Met62Argfs*8), was detected in our patient (proband) (Figure 1B–D). For the studied gene, the mean coverage was 418.09×; the detailed coverage was >40× for 100.00% of the test region sequences. The deletion caused a frame shifting change with Metionine -62 replaced by Arginine and created new reading frame ending at a stop at position 8. The variant detected in the study has not yet been registered in the HGMD^®^ Professional 2019.4, ClinVar or Leiden Open Variation Database databases. Heterozygous c.185delT mutation was not confirmed in both the clinically unaffected parents, as they were dead at the time of the study.

### 3.2. Light Microscopy Data

**Figure 2:**
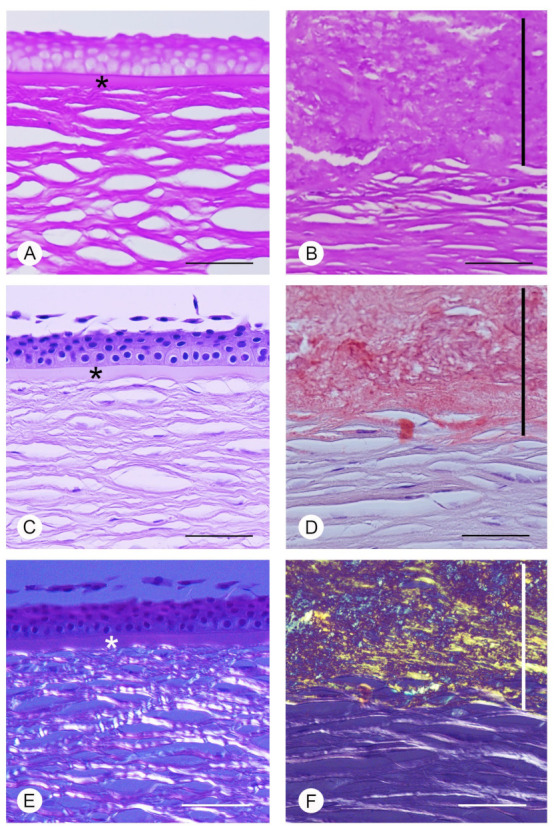
Raman spectroscopy in a corneal dystrophy. PAS and Congo Red-stained normal and GDCD cornea. (**A**) In normal cornea, with PAS stain a mild positivity is present in the superficial epithelial cells, in the Bowman’s layer (*) and in the stroma. (**B**) In GDCD cornea, PAS^+^ deposits (black vertical line) are present over the anterior stroma. (**C**) In normal cornea, between the epithelium and the anterior stroma, an evident Bowman’s layer (*) is present. (**D**) In GDCD cornea, note the presence of irregular deposits (black vertical line) over the anterior stroma, exhibiting distinctive brick red stain with Congo red stain. Bowman’s layer cannot be appreciated. (**E**) Under polarized light, no Congo red positive material can be demonstrated in the normal cornea. * = Bowman’s layer. (**F**) In GDCD cornea, a characteristic apple green birefringence under polarized light (white vertical line) is present (Scale bar: 100 μm).

In the PAS stained normal cornea (Figure 2A), a mild positivity was present in the superficial epithelial cells, in the Bowman’s layer and in the stroma. On the contrary, in the GDCD cornea (Figure 2B), PAS positive material was present over the anterior stroma. In the Congo Red-stained normal cornea, between the epithelium and the anterior stroma, an evident Bowman’s layer was present (Figure 2C). In the GDCD cornea stained with Congo Red, the Bowman’s layer was missing and irregular deposits, exhibiting distinctive brick red stain, were instead present over the anterior stroma (Figure 2D). When the normal cornea was examined with polarized light, no Congo Red positive material could be demonstrated (Figure 2E); instead in the GDCD cornea, the characteristic apple green birefringence was evident over the anterior stroma (Figure 2F).

### 3.3. Raman Spectroscopy Data

**Figure 3:**
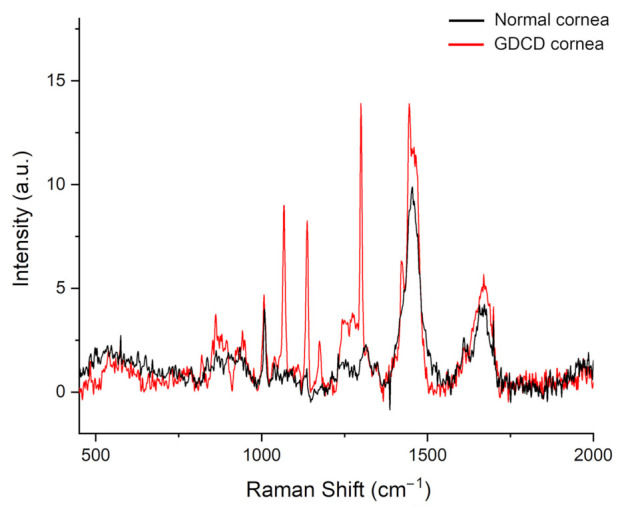
Raman spectroscopy in a corneal dystrophy. Average Raman spectrum of normal (black line) and GDCD (red line) cornea, in the spectral range of 450–2000 cm^−1^.

The average Raman spectra of cornea from normal and GDCD cornea in the range of 450–2000 cm^−1^ was reported in Figure 3. The spectra confirmed the main typical protein vibrational modes, which derive from the polypeptide backbone (amide bands) and from aromatic and non-aromatic amino acid residue side chains.

It was possible to observe differences in peak position, width, and intensity between the two spectra. In particular, in the GDCD cornea, several peaks located in the range 855–1670 cm^−1^ were higher when compared to the normal cornea. Furthermore, in the GDCD cornea, three peaks, absent in the normal cornea, were demonstrated at the range ~1068 cm^−1^, 1138 cm^−1^, and 1421 cm^−1^. The tentative assignment of the main vibrational bands was stated on the basis of the literature and the aforementioned differences between GDCD and normal cornea were summarized in Table 1.

## 4. Discussion

Corneal dystrophies are a complex group of inherited diseases, generally able to impair vision owing to the presence of corneal opacities. GDCD is included among IC3D category 1 dystrophies, as its involved gene (*TACSTD2*) and location of mutation (chromosome 1p32) were identified [8].

As to the mutations observed, p.Glu118Ter (Q118X) was found to be the most common [6], even if many other mutations were identified in patients from different countries [5,6,12,37,38]. The genetic analysis of our Polish patient showed for the first time the presence of a variant not registered in the current databases, that is a frameshift mutation c.185delT, resulting in a TACSTD2 molecule containing an arginine instead of a methionine at the 62nd amino acid. The substitution of the non-polar amino acid methionine by the basic polar arginine changes the structure of the protein encoded [6], which loses its role in the stabilization of claudin-1 and -7, and integrins, causing a dysfunction in the corneal epithelial barrier [38].

As a consequence, lactoferrin and lysozyme, major components of the tears, can penetrate through the corneal epithelium and the Bowman’s layer, and accumulate in the anterior stroma as amyloid deposits [6]. Our morphological data, even if not compared with positive histologic control for amyloid, but supported by references on the topic [6,12,38], confirmed the positivity of the corneal deposits for PAS and Congo Red stains and their birefringence under the polarized microscope, typical of amyloid.

Raman spectroscopy provides data about vibrational, rotational, and other low frequency transitions in molecules. In pathological events, it is possible to observe tissue changes, which exert their effects on molecular motions [39]. Therefore, it was expected that Raman spectroscopy could be used to provide adequate information on biochemical changes in subjects with GDCD, when compared to normal cornea.

From the acquired spectra, our attention was focused on the spectral range 450–1900 cm^−1^, where proteins, lipids, carbohydrates, nucleic acids, and other molecules are present [28,40,41]. Peculiar differences in peak position, width, and intensity were demonstrated and, more importantly, in GDCD average spectrum peaks not present in the normal cornea were evident.

As to the peaks present in both the specimens, even with differences in their height, we found a prominent shift for the peaks located at ~855 cm^−1^ and at ~1173 cm^−1^ and assigned to tyrosine [28,32,34], an amino acid present in amyloid deposits [41].

The peaks intensity of 886 cm^−1^ and 940 cm^−1^, related to tryptophan [25,33] and proline/valine, respectively [28], were higher in GDCD when compared to the control cornea. Even if these biomolecules are normally present in the cornea [42,43], the increased intensity of both the above peaks in the Raman spectra could be related to polyglutamine aggregates, formed from monomers with different length, and present in amyloid fibrils [41,44].

Particular relevance can be attributed to the peaks at ~1200–1300 cm^−1^ related to the Amide III band [34]. They were particularly high in GDCD cornea when compared to normal and are considered typical of the amyloid material [45], and in particular to lactoferrin [36].

As to the band demonstrated at ~1440 cm^−1^, particularly evident in both normal and dystrophic cornea, its molecular origin can be referred to the CH_2_ peak, typical of either collagenous or non-collagenous organic molecules [46], such as lactoferrin [35,45].

The large band peaking at ~1650 cm^−1^ (Amide I band) can be related to the presence of collagen and proteins [46,47], whose content is increased in GDCD cornea [38].

Finally, in addition to the differences in the intensity of peaks present in both normal and GDCD cornea, in GDCD cornea, we revealed some other peaks not evident in the normal cornea and located at ~1068 cm^−1^, at ~1138 cm^−1^, and ~1421 cm^−1^. As to their meaning, the analysis of the existing literature showed that similar peaks were observed in a single study considering histological sections from cervical tissue in which paraffin wax was not entirely removed [48]. However, in our study, at least three sections for each cornea were examined with superimposable results, both control and GDCD cornea were processed simultaneously according to an identical protocol, xylene was used for an appropriate time longer than the cited paper, no impairment in staining was observed [48], so that it seems difficult to propose that paraffin wax was still present only in a single specimen (GDCD cornea). Therefore, we are of the opinion that the above indicated peaks, for which no convincing correspondence was found in the current literature, as indicated in Table 1, can be probably related to biochemical changes of the corneal tissue in the disease.

Limits of our study can be considered to be the evaluation of a single patient, even if the disease is rare, and the absence of a positive control for amyloid when Raman spectroscopy was considered. However, as mentioned above, the comparison with the existing literature [32,34,46] supported our data.

## 5. Conclusions

Our study demonstrated a new mutation of the *TACSTD2* gene, confirmed the structural changes of the cornea in GDCD, and showed, for the first time, peculiar changes in GDCD cornea using Raman spectroscopy, related to the presence of amyloid deposits. The overall results, which must be confirmed by the study in a larger population, suggest a possible use of the Raman technique to perform in vivo measurements in other corneal diseases, with enormous implications in facilitating early specific diagnosis, reducing biopsies and/or any invasive procedure. Therefore, it can be suggested either as a screening tool or a useful procedure for monitoring disease progression, thus providing clinicians with valuable information.

## Figures and Tables

**Table 1:** Tentative assignment of the main vibrational bands based on the literature.

Frequency (cm^−1^)	Tentative Assignment	Normal Cornea	GDCD Cornea	References
855	Tyrosine	visible	visible	[32]
886	Tryptophan	visible	visible	[25,33]
940	Proline, valine	visible	visible	[28]
1005	Phenylalanine	visible	visible	[28,32]
1068	Unassigned	-----	visible	-----
1138	Unassigned	-----	visible	-----
1173	Tyrosine	visible	visible	[34]
1200–1300	Amide III band	visible	visible	[34]
1421	Unassigned	------	visible	-----
1440	Vibrational stretching CH_2_	visible	visible	[35]
1650	Amide I band	visible	visible	[35,36]

## Data Availability

The data presented in this study are available on request to the corresponding author.
